# Effects of Statins on All-Cause Mortality in Patients with Breast Cancer: A Population-Based Study

**DOI:** 10.3390/biomedicines13071556

**Published:** 2025-06-25

**Authors:** Ching-Feng Cheng, Chao-Hsu Li, Joshua Wang, Kuo-Cheng Lu, Kuo-Wang Tsai

**Affiliations:** 1 Department of Pediatrics, Taipei Tzu Chi Hospital, Buddhist Tzu Chi Medical Foundation, New Taipei City 23142, Taiwan; chengcf@tzuchi.com.tw; 2 School of Medicine, Tzu Chi University, Hualien 97004, Taiwan; xd710212@tzuchi.com.tw; 3 Division of General Surgery, Department of Surgery, Taipei Tzu Chi Hospital, Buddhist Tzu Chi Medical Foundation, New Taipei City 23142, Taiwan; 4 Department of Research, Taipei Tzu Chi Hospital, Buddhist Tzu Chi Medical Foundation, New Taipei City 23142, Taiwan; j3.reilly@qut.edu.au; 5 Division of Nephrology, Department of Medicine, Taipei Tzu Chi Hospital, Buddhist Tzu Chi Medical Foundation, New Taipei City 23142, Taiwan; 6 Department of Nursing, Cardinal Tien Junior College of Healthcare and Management, New Taipei City 23143, Taiwan

**Keywords:** all-cause mortality, breast cancer, survival, statin

## Abstract

This retrospective cohort study investigated the effects of statin use on 5-year clinical outcomes, particularly all-cause mortality, in patients with breast cancer. Clinical data of 971,808 patients who received a diagnosis of breast cancer between 2010 and 2020 were collected from the TriNetX platform. Eligible patients were classified as statin users (98,761) or nonusers (691,644). Statin use was defined by a prescription of statins being given within 3 years after breast cancer diagnosis. All-cause mortality and cardiovascular incidence were evaluated from Aalen–Johansen cumulative incidence curves. After 1:1 propensity score matching, all-cause mortality outcomes were analyzed in terms of hazard ratios and risk ratios. Our studies revealed that the risk of all-cause mortality was lower in statin users than in nonusers (hazard ratio: 0.798; risk ratio: 0.721; *p* < 0.001). Subgroup analysis revealed that the protective effect of statins against all-cause mortality was more pronounced in older patients; those with a higher body mass index; and those with higher cholesterol, triglyceride, or low-density lipoprotein levels. The effects were prominent also in patients with estrogen receptor-negative or progesterone receptor-negative tumors. Statin use was associated with improved survival in patients with breast cancer, particularly older patients, those with hormone receptor-negative tumors, and those with metabolic dysregulation. Our findings indicate a possible link between statin use and reduced mortality in breast cancer patients, warranting further investigation in prospective controlled studies.

## 1. Introduction

Breast cancer is one of the most common malignancies worldwide; its progression is influenced by genetic, metabolic, and environmental factors [1,2]. It is a heterogeneous disease classified into various molecular subtypes, such as luminal A breast cancer, luminal B breast cancer, HER2-enriched breast cancer, and triple-negative breast cancer (TNBC) [3]. These subtypes differ in their biological behaviors and therapeutic responses [4]. Post-treatment disease progression varies markedly across the subtypes [5]. The most aggressive subtype of breast cancer is TNBC, characterized by the lack of estrogen receptor (ER), progesterone receptor (PR), and HER2 expression. TNBC carries a high risk of early progression and has limited options for targeted therapy, resulting in the worst prognosis [6]. Therefore, developing new therapeutic strategies for different breast cancer subtypes would benefit patients.

Statins, widely known for their cholesterol-lowering effects, have recently gained attention in breast cancer research for their potential to regulate key oncogenic pathways [7,8,9]. These drugs are generally classified into two types on the basis of solubility: lipophilic statins (e.g., simvastatin, fluvastatin, atorvastatin, and pitavastatin) and hydrophilic statins (e.g., pravastatin and rosuvastatin) [10]. Statins exert their anticancer effects primarily by inhibiting 3-hydroxy-3-methylglutaryl-CoA reductase, thereby disrupting cholesterol biosynthesis and altering key cellular processes such as proliferation, apoptosis, and metastasis [11,12,13,14]. Notably, the overexpression of 3-hydroxy-3-methylglutaryl-CoA reductase is strongly associated with increased tumor aggression and poor prognosis [15]. Statins suppress tumor progression mainly by inhibiting cell survival-related signaling cascades, such as the phosphoinositide 3-kinase/protein kinase B (AKT) and mitogen-activated protein kinase/extracellular signal-regulated kinase pathways, thus inducing cell cycle arrest and reducing tumor cell proliferation [10,16,17,18]. In addition, statins suppress the prenylation of RHO and RAC proteins, regulating cytoskeletal reorganization, cell motility, and adhesion and thereby reducing metastatic potential [13,19]. Emerging research suggests that statins modulate the tumor microenvironment by alleviating inflammation and enhancing immune surveillance, thus improving cancer outcomes [20,21]. In summary, statins potentially improve breast cancer outcomes by modulating cancer cell growth, metastasis, and the tumor microenvironment.

The effects of statins on breast cancer progression remain inconclusive and depend on the breast cancer subtype. In luminal A and luminal B subtypes, which rely on ER signaling, statins may affect ER activity by altering the composition of lipid rafts [22,23,24]. Previous studies revealed that the use of statins to inhibit cholesterol biosynthesis enhanced the efficacy of AKT inhibitors in breast cancer [16,25,26]. However, a study demonstrated that neither ER-positive breast cancer cells nor ER-positive organoids are sensitive to the combination of AKT inhibitors and pitavastatin [16]. In HER2-enriched breast cancer, statins may disrupt lipid raft structures that support the localization of HER2 receptor, inhibiting HER2-related signaling, potentiating HER2-targeted therapy (e.g., trastuzumab), and delaying resistance development [22]. Guo et al. reported that the effects of statins were relatively pronounced in hormone receptor-positive/HER2-negative breast cancer [27]. Collectively, these findings indicate that the efficacy of statins in treating breast cancer remains questionable, necessitating further research. Although epidemiological and preclinical data suggest that statins exert anticancer effects, clinical trial results remain inconclusive [28]. Further validation through real-world data is necessary, particularly to clarify the efficacy of statins in treating various breast cancer subtypes and the therapeutic benefits of these drugs for high-risk populations, such as patients with obesity or metabolic syndrome. To address these research gaps, the present retrospective study investigated the effect of statins on breast cancer progression and explored the potential applications of these drugs in personalized therapy.

## 2. Methods

### 2.1. Data Source

The study protocol was approved by the institutional review board of our hospital. Patient data were extracted from the TriNetX Global Collaborative Network, which contains the data of 163,457,967 patients across 140 health-care centers worldwide. TriNetX analyses anonymized patient data in compliance with the Health Insurance Portability and Accountability Act and were therefore exempt from institutional Human Research Ethics review processes. This study included 971,808 patients (age: 18–90 years) who received a diagnosis of breast cancer (*International Classification of Diseases*, *Tenth Revision*, *Clinical Modification* code: C50) between 1 January 2010 and 1 January 2020. They were stratified into statin users and nonusers.

### 2.2. Data Analysis

Statin use was defined by the presence of a prescription of any statin (atorvastatin, rosuvastatin, simvastatin, pravastatin, lovastatin, pitavastatin, or fluvastatin) being given within 3 years after the first recorded diagnosis of breast cancer. Patients were only included in the statin use group if they had at least three prescriptions of a statin. Patients in the statin nonusers group had never been prescribed any of the above statins.

To minimize confounding effects, 1:1 propensity score matching was performed, with adjustments for age at initial breast cancer diagnosis, sex, race, diabetes mellitus (DM), kidney disease, and hypertension. Thus, two well-matched cohorts of 93,435 patients each were formed. The following 5-year clinical outcomes were assessed: all-cause mortality and cardiovascular events (*International Classification of Diseases*, *Tenth Revision*, *Clinical Modification* codes: I21, I26, I47.2, I48, I49.0, I50.21, I50.23, I50.31, I50.33, and I63). Patients having any of these outcomes before receiving a breast cancer diagnosis were excluded from the risk analysis for that specific outcome. In the Kaplan–Meier survival analysis, a time window was applied to include patients who made ≥1 outpatient visit after 5 years from the first diagnosis of breast cancer. This ensured that data from both groups were not censored due to having their last clinical fact recorded during the outcome period. Data collection was completed on 7 March 2025.

### 2.3. Statistical Analysis

All statistical analyses were conducted using the analytical tools available within the TriNetX platform. Aalen–Johansen cumulative incidence curves were used to assess all-cause mortality and the incidence of cardiovascular events. Kaplan–Meier survival analysis was applied to estimate survival probabilities over a 5-year follow-up period, while Cox proportional hazards regression was used to calculate hazard ratios (HRs) for clinical outcomes between groups during this timeframe. The Risk Ratio (RR) compares the probability of all-cause mortality in statin users to that in nonusers, indicating how much the risk changes with statin use. The Odds Ratio (OR) compares the odds of all-cause mortality between statin users and nonusers, and is commonly applied in case–control studies and logistic regression models.

## 3. Results

### 3.1. Clinical Characteristics of Patients Stratified by Statin Use

**Figure 1:**
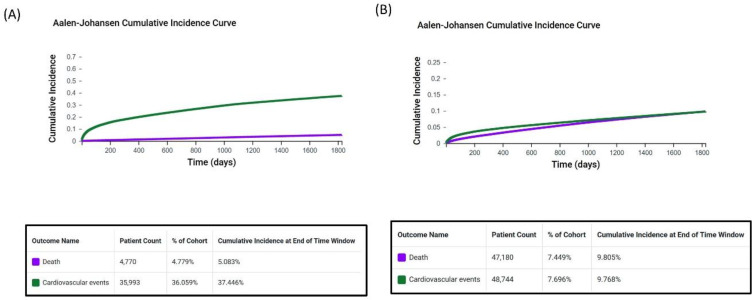
Cumulative incidence of all-cause mortality and cardiovascular events in patients with breast cancer. The figures depict 5-year cumulative incidence in (**A**) statin users and (**B**) nonusers.

From the TriNetX data, we identified 971,808 patients (age: 18–90 years) who received a breast cancer diagnosis during the study period. The statin user and nonuser groups had 104,754 and 692,146 patients, respectively. Among statin users, the cumulative incidence of cardiovascular events was significantly higher than the rate of mortality (37.446% vs. 5.083%; Figure 1). Among nonusers, the incidence of cardiovascular events was similar to the rate of mortality (9.768% vs. 9.805%). These results highlight a difference in outcomes between the two groups. Compared with nonusers, statin users tended to be older at the index date (67.8 ± 10.6 vs. 58.6 ± 14.1 years) and have a higher body mass index (30.3 ± 7.2 vs. 27.7 ± 6.8 kg/m^2^). The following comorbidities were more prevalent among statin users than among nonusers (Table 1): DM (22.3% vs. 3.1%), kidney disease (7.3% vs. 1.5%), and hypertension (46.6% vs. 10.5%). Furthermore, blood levels of cholesterol, low-density lipoprotein (LDL), and high-density lipoprotein were significantly lower in statin users than in nonusers, whereas those of triglycerides were significantly higher in statin users than in nonusers (Table 1).

### 3.2. Effects of Statin Use on Survival

**Figure 2:**
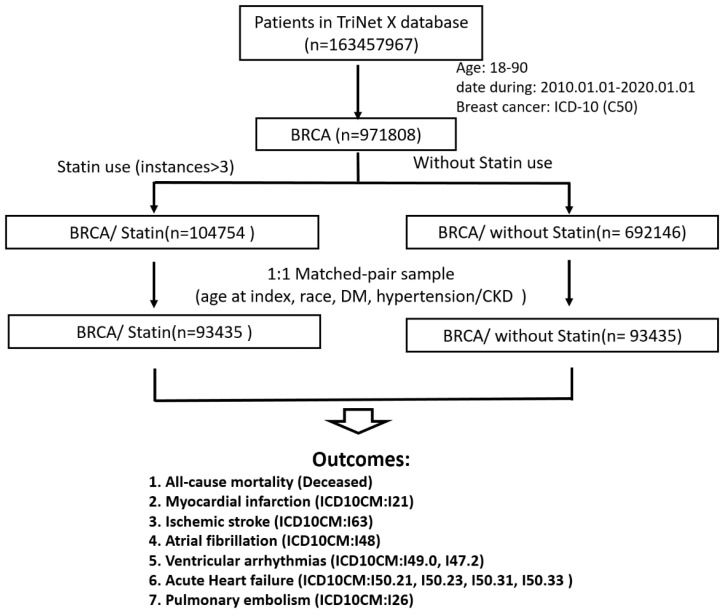
Flowchart depicting the study pipeline. A cohort of patients with breast cancer was selected from the TriNetX platform. The patients were stratified by statin use into two groups (statin users and nonusers) and subjected to 1:1 propensity score matching.

After 1:1 propensity score matching for age, race, DM, hypertension, and kidney disease, each of the two matched groups comprised 93,435 patients (Figure 2). The following clinicodemographic characteristics were well balanced between statin users and nonusers after propensity score matching (Table 1): age on the index date (67.6 ± 10.7 vs. 68.0 ± 10.9 years), race, DM prevalence (17.6% vs. 16.6%), kidney disease prevalence (6.4% vs. 6.3%), and hypertension prevalence (43.4% vs. 43.6%). 

**Figure 3:**
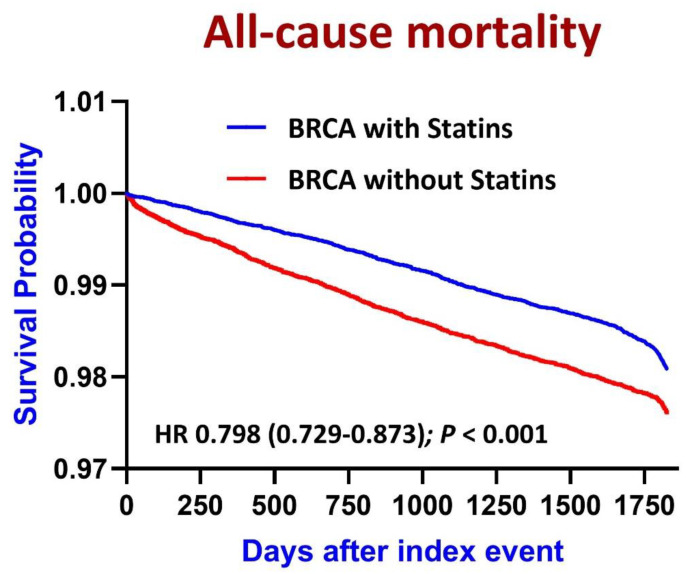
Kaplan–Meier curves depicting 5-year all-cause mortality rates in patients with breast cancer (BRCA) stratified by statin use.

Statin use was significantly associated with a reduced risk of all-cause mortality in patients with breast cancer (risk ratio [RR]: 0.721; 95% confidence interval [CI]: 0.705–0.738; *p* < 0.001; Table 2). Moreover, Kaplan–Meier survival analysis revealed that statin use was associated with a relatively long survival (HR: 0.798; 95% CI: 0.729–0.873; *p* < 0.001; Figure 3). These findings suggest that statins confer their protective effects by reducing the risk of all-cause mortality. However, statin use was significantly associated with elevated risks of cardiovascular events such as myocardial infarction (RR: 4.390; 95% CI: 4.105–4.694), ischemic stroke (RR: 4.250; 95% CI: 4.009–4.505), atrial fibrillation (RR: 2.272; 95% CI: 2.190–2.358), ventricular arrhythmias (RR: 2.644; 95% CI: 2.560–2.731), acute heart failure (RR: 3.502; 95% CI: 3.305–3.711), and pulmonary embolism (RR: 1.864; 95% CI: 1.757–1.978).

### 3.3. Subgroup Analysis of All-Cause Mortality

**Figure 4:**
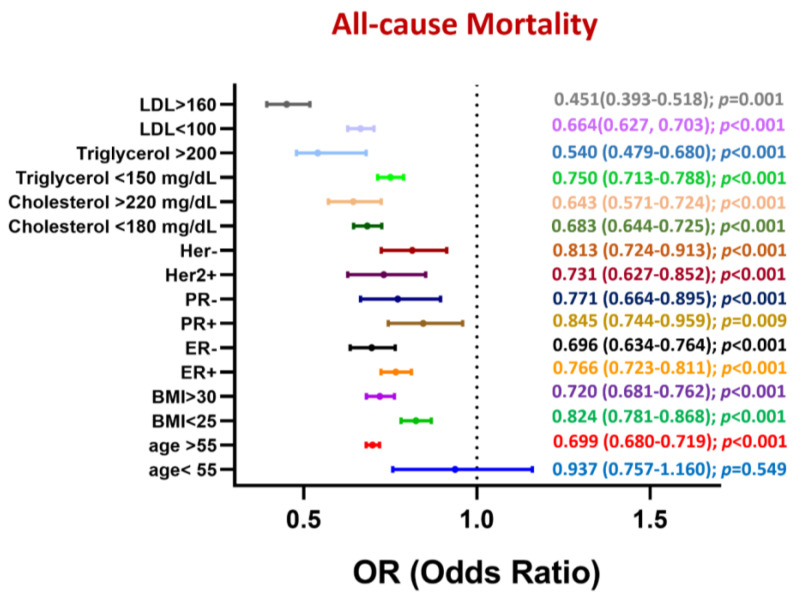
Subgroup analysis of 5-year mortality rate in patients with breast cancer stratified by statin use.

A detailed subgroup analysis of 5-year all-cause mortality in patients with breast cancer revealed that statins significantly reduced the risk of all-cause mortality across all subgroups (Figure 4). However, statins exerted no significant effect on this risk in younger patients (odds ratio [OR]: 0.937; 95% CI: 0.757–1.160; *p* = 0.549). The protective effects of these drugs were more pronounced in older patients (OR: 0.699; 95% CI: 0.680–0.719; *p* < 0.001) and those with a higher body mass index (>30 kg/m^2^; (OR: 0.720; 95% CI: 0.681–0.762; *p* < 0.001). The statin-induced reduction in the risk of all-cause mortality was larger in patients with ER-negative breast cancer (OR: 0.696; 95% CI: 0.634–0.764; *p* < 0.001) and patients with PR-negative breast cancer (OR: 0.771; 95% CI: 0.664–0.895; *p* < 0.001) than in their counterparts with the corresponding conditions. Furthermore, the protective benefits of statins were greater in patients with higher blood levels of cholesterol (>220 mg/dL; OR: 0.643; 95% CI: 0.571–0.724; *p* < 0.001), triglycerides (>200 mg/dL; OR: 0.540; 95% CI: 0.479–0.680; *p* < 0.001), and LDL (>160 mg/dL; OR: 0.451; 95% CI: 0.393–0.518; *p* < 0.001). These findings highlight the efficacy of statin use in improving survival in patients with breast cancer, particularly older patients and those with high triglyceride, cholesterol, or LDL levels.

## 4. Discussion

Our findings revealed that statin use in patients with breast cancer was associated with a significant reduction in the risk of all-cause mortality (HR: 0.798; 95% CI: 0.729–0.873; *p* < 0.001). This finding aligns with those of epidemiological studies highlighting an association between statin use and improved survival in patients with breast cancer [29,30,31]. Statins exert antitumor effects by inhibiting the mevalonate pathway, thereby disrupting key oncogenic signaling cascades such as the phosphoinositide 3-kinase/AKT and RAS/mitogen-activated protein kinase pathways, ultimately suppressing tumor proliferation and metastasis [13,17,18,19,32]. In addition to suppressing oncogenic pathways, statins may decelerate cancer progression by altering the tumor microenvironment. Li et al. demonstrated that lovastatin markedly downregulated the paclitaxel-induced expression of programmed death-ligand 1 and increased the activity of CD8+ T cells, thus supporting paclitaxel-mediated inhibition of tumor growth [33]. Previous review papers indicated that statins may improve breast cancer outcomes by promoting tumor cell apoptosis and reducing metastasis [34,35,36]. However, their long-term effects and optimal use remain uncertain. Consequently, the efficacy of statins may vary depending on the patient’s physical condition, breast cancer subtype, the specific statin used, and the timing of administration [34].

Guo et al. reported that statins significantly reduced the risk of cancer-specific mortality, particularly in patients with hormone receptor-positive and HER2-negative breast cancer [27]. Similarly, Murto et al. indicated that statins reduced the risk of mortality in patients with ER-positive breast cancer [37]. Corroborating these findings, Scott et al. reported a significant association between statin use and reduced mortality risk in patients with breast cancer, particularly those with ER-positive tumors, postmenopausal women, and those with advanced-stage disease [38]. Statins may disrupt ER signaling by inhibiting the mevalonate pathway, thereby enhancing the efficacy of endocrine therapy in hormone receptor-positive breast cancer [22,39]. However, the present study revealed that both patients with ER-negative or PR-negative tumors benefited more from statin use than did their counterparts, implying that statins exert direct antitumor effects independent of hormone signaling. A study reported that inhibiting cholesterol biosynthesis with statins enhanced the efficacy of AKT inhibitors in treating TNBC xenografts and patient-derived ER-negative breast cancer organoids [16]. However, ER-positive breast cancer cells and organoids did not respond to the combination of an AKT inhibitor and pitavastatin. Statins suppress the HER2-induced AKT and extracellular signal-regulated kinase pathway by altering the localization of RAC1, thereby improving prognosis in patients with HER2-positive breast cancer [40]. We found that patients with HER2-positive tumors benefited more from statin use than did those with HER2-negative tumors.

Our results indicated that statin users were older and had a higher BMI compared to nonusers, which likely reflects a correlative observation rather than a causal relationship. It is likely that this pattern arises due to confounding by indication, as individuals who are older or have higher BMI are at greater risk for cardiovascular diseases and are thus more likely to be prescribed statins. Therefore, the difference in age and BMI between statin users and nonusers probably reflects underlying clinical risk profiles rather than an effect of statin use itself. In addition, the cumulative incidence of cardiovascular events was higher than the rate of mortality among statin users. However, among nonusers, the cumulative incidence of cardiovascular events was similar to the rate of mortality. Therefore, patients with breast cancer who were prescribed statin therapy might have had an elevated cardiovascular risk at baseline, likely attributable to comorbidities such as hypertension, DM, and obesity, which were more prevalent among statin users than among nonusers. Evidence suggests that the presence of metabolic disorders often confounds the benefits of statin use, making it challenging to identify the direct effects of statins on breast cancer outcomes [41]. Although statin use is associated with a reduced risk of all-cause mortality, it is also associated with elevated risks of cardiovascular events, such as myocardial infarction, ischemic stroke, and acute heart failure. This association may be attributable to the higher baseline cardiovascular risk in statin users than in nonusers. Thus, clinicians must carefully assess cardiovascular risk in patients with breast cancer receiving statin therapy.

This study yielded several key findings regarding statin use in patients with breast cancer. Unlike other studies suggesting broad protective effects across all age groups, our study revealed no significant survival benefits of statins in younger patients (OR: 0.937; 95% CI: 0.757–1.160; *p* = 0.549). The protective effects of statins were relatively pronounced in patients with a high risk of metabolic syndrome, particularly those with high blood levels of cholesterol (>220 mg/dL), triglycerides (>200 mg/dL), and LDL (>160 mg/dL). These findings suggest that statins are particularly beneficial for older patients and those with severe lipid metabolism disorders, supporting the notion that cholesterol biosynthesis plays a key role in the progression of breast cancer.

Our study has several limitations. First, statin users had a higher baseline cardiovascular risk than did nonusers, making it challenging for us to determine whether the observed reduction in the risk of all-cause mortality was due to statin use itself or indirect factors such as improved overall medical care. Second, we did not differentiate between lipophilic statins and hydrophilic statins. Although we did not perform subgroup analyses based on statin lipophilicity, prior studies have suggested that lipophilic statins may exert stronger anticancer effects than hydrophilic statins due to their enhanced ability to penetrate extrahepatic tissues [25,42,43,44]. Third, precise data on the dose and duration of statin use were unavailable. Finally, the possibility of a selection bias cannot be ignored, given that the prevalence of metabolic comorbidities and the likelihood of receiving long-term medical care were relatively high among statin users; this might have led to the early detection of cancer progression in statin users and enabled them to have access to advanced treatment options, partially explaining the observed survival benefits. In addition, a review paper reported that statins reduce breast cancer recurrence rates when administered after diagnosis [34]. Although recurrence-related survival outcomes could offer further insights, the lack of recurrence-specific data within the TriNetX platform limits our ability to conduct such analyses. Future studies with access to detailed recurrence and progression data are warranted. 

## 5. Conclusions

This study adds to the growing body of evidence suggesting that statins offer survival benefits for patients with breast cancer, particularly older patients and those with metabolic dysregulation. However, the increased cardiovascular risk in statin users highlights the need for personalized therapeutic strategies that balance the benefits of improved survival with the management of cardiovascular risk.

## Figures and Tables

**Table 1:** Patients’ baseline characteristics before and after propensity score matching.

Characteristic Name	Before Propensity Score Matching				After Propensity Score Matching			
	Patients with Statins (*n* = 104,754)	Patients without Statins (*n* = 692,146)	*p* Value	Std diff	Patients with Statins (n = 93,435)	Patients without Statins (*n* = 93,435)	*p* Value	Std diff
**Basic demographics (%)**								
Age at Index, mean (mean+/−SD)	67.8 +/− 10.6	58.6 +/− 14.1	<0.001	0.74	67.6 +/− 10.7	68.0 +/− 10.9	<0.001	0.038
White (%)	70.80%	53.60%	<0.001	0.36	71.20%	71.60%	0.045	0.009
Black or African American (%)	14.80%	7.00%	<0.001	0.25	13.70%	13.10%	<0.001	0.019
Asian (%)	3.90%	5.00%	<0.001	0.05	3.90%	3.90%	0.559	0.003
**Laboratory, Mean ± SD**								
Glucose [Mass/volume] in Serum, Plasma, or Blood	118.1 +/− 47.1	105.4 +/− 34.3	<0.001	0.31	116.0 +/− 45.3	113.3 +/− 43.4	<0.001	0.06
Alanine aminotransferase [Enzymatic activity/volume] in Serum, Plasma, or Blood	23.8 +/− 27.2	26.8 +/− 57.4	<0.001	0.07	23.9 +/− 26.7	27.3 +/− 63.1	<0.001	0.071
Aspartate aminotransferase [Enzymatic activity/volume] in Serum or Plasma	25.3 +/− 32.3	31.6 +/− 94.5	<0.001	0.09	25.3 +/− 30.8	33.3 +/− 95.8	<0.001	0.112
Alkaline phosphatase [Enzymatic activity/volume] in Serum, Plasma, or Blood	84.3 +/− 47.7	94.2 +/− 97.8	<0.001	0.13	84.1 +/− 48.1	97.5 +/− 92.6	<0.001	0.182
Lactate dehydrogenase [Enzymatic activity/volume] in Serum or Plasma	248.1 +/− 243.6	270.7 +/− 360.5	<0.001	0.074	247.6 +/− 247.8	292.2 +/− 380.9	<0.001	0.139
Bilirubin.Total [Mass/volume] in Serum, Plasma, or Blood	0.5 +/− 0.7	0.6 +/− 1.1	<0.001	0.084	0.5 +/− 0.6	0.6 +/− 1.2	<0.001	0.096
Albumin [Mass/volume] in Serum, Plasma, or Blood	4.0 +/− 0.5	4.0 +/− 0.6	<0.001	0.025	4.0 +/− 0.5	3.9 +/− 0.6	<0.001	0.278
Cholesterol [Mass/volume] in Serum or Plasma	183.5 +/− 47.2	196.3 +/− 41.3	<0.001	0.287	185.1 +/− 47.0	192.6 +/− 43.7	<0.001	0.166
Cholesterol in LDL [Mass/volume] in Serum or Plasma	101.3 +/− 39.3	111.7 +/− 33.3	<0.001	0.287	102.5 +/− 39.3	109.6 +/− 34.7	<0.001	0.192
Cholesterol in HDL [Mass/volume] in Serum or Plasma	54.1 +/− 18.7	60.0 +/− 21.1	<0.001	0.296	54.6 +/− 18.8	57.6 +/− 21.7	<0.001	0.15
Triglyceride [Mass/volume] in Serum, Plasma, or Blood	138.4 +/− 83.0	114.6 +/− 70.5	<0.001	0.308	137.8 +/− 83.1	122.9 +/− 74.3	<0.001	0.189
Hemoglobin A1c/Hemoglobin.total in Blood	6.8 +/− 1.5	6.1 +/− 1.3	<0.001	0.494	6.7 +/− 1.5	6.4 +/− 1.5	<0.001	0.194
BMI	30.3 +/− 7.2	27.7 +/− 6.8	<0.001	0.364	30.1 +/− 7.1	28.8 +/− 7.2	<0.001	0.175
**Diagnosis (%)**								
Diabetes Mellitus	22.30%	3.10%	<0.001	0.602	17.60%	16.60%	<0.001	0.027
Acute Kidney Failure and Chronic Kidney Disease	7.30%	1.50%	<0.001	0.288	6.40%	6.30%	0.276	0.005
Hypertensive Diseases	46.60%	10.50%	<0.001	0.871	43.40%	43.60%	0.253	0.005

**Table 2:** Associations of statin use with various outcomes in patients with breast cancer.

Outcomes	Groups	Cases Followed	Incident Cases	Risk (%)	Risk Ratio	Odds Ratio	Risk Difference (*p* Value)
**All-cause Mortality**	with statins	93,236	10,412	11.2	0.721 (0.705, 0.739)	0.686 (0.668, 0.705)	<0.001
	without statins	92,578	14,332	15.5			
**Myocardial Infarction**	with statins	90,661	4454	4.9	4.390 (4.105, 4.694)	4.565 (4.264, 4.887)	<0.001
	without statins	92,569	1036	1.1			
**Ischemic Stroke**	with statins	89,437	5653	6.3	4.250 (4.009, 4.505)	4.469 (4.210, 4.744)	<0.001
	without statins	92,048	1369	1.5			
**Atrial Fibrillation**	with statins	86,617	8473	9.8	2.272 (2.190, 2.358)	2.410 (2.317, 2.507)	<0.001
	without statins	88,226	3798	4.3			
**Ventricular Arrhythmias**	with statins	85,840	11,919	13.9	2.644 (2.560, 2.731)	2.909 (2.808, 3.013)	<0.001
	without statins	89,702	4711	5.3			
**Acute Heart Failure**	with statins	91,903	4999	5.4	3.502 (3.305, 3.711)	3.646 (3.436, 3.869)	<0.001
	without statins	92,775	1441	1.6			
**Pulmonary Embolism**	with statins	92,007	3043	3.3	1.864 (1.757, 1.978)	1.894 (1.782, 2.013)	<0.001
	without statins	92,281	1637	1.8			

## Data Availability

Data is contained in the article.
